# Meta-analysis of cancer incidence in children born after assisted reproductive technologies

**DOI:** 10.1038/sj.bjc.6602838

**Published:** 2005-10-18

**Authors:** S Raimondi, P Pedotti, E Taioli

**Affiliations:** 1 Fondazione Policlinico IRCCS-Molecular and Genetic Epidemiology Unit, via Pace 9, Milano 20122, Italy

**Keywords:** cohort study, *in vitro* fertilisation, childhood chronic diseases

## Abstract

A meta-analysis was performed on 11 cohort studies of Assisted Reproductive Technologies (ART) and subsequent childhood cancer, published up to February 2005, which reported comparable, nonoverlapping data, and then restricted to eight studies which presented a similar research design. The overall Standardised Incidence Ratio was 1.33 (95% CI 0.62–2.85), and 0.77 (95% CI 0.41–1.42) when the analysis was restricted to eight studies. No evidence of publication bias was observed for the overall analysis. The data are consistent with a lack of increase in risk of childhood cancer, though the amount of data on ART and cancer is still limited; larger multicentric studies as well as a pooled analysis on the available data are warranted.

Since the first child was born after *In Vitro* Fertilisation (IVF) in 1978, several studies have been conducted on the possible consequences of Assisted Reproductive Technologies (ART), which include standard IVF, Gamete Intra-Fallopian Transfer (GIFT) and Intra-Cytoplasmatic Sperm Injection (ICSI). Most papers on the consequences of ART concentrated on short-term outcomes, such as perinatal mortality, multiple pregnancies, weight at birth and malformations (reviewed in Schieve *et al*, 2004 and Hansen *et al*, 2005), while few studies have considered the long-term effects of these techniques.

Now that several children born after ART have reached adolescence, it is useful to study the possible long-term consequences of this procedure, such as cancer incidence. Cases of cancer in children born after ART have been reported (White *et al*, 1990; Toren *et al*, 1995; Rizk *et al*, 2000; Cruysberg *et al*, 2002; Lee *et al*, 2004); the hypotheses behind a possible association between ART and cancer could be the repeated hormonal exposure and/or the epigenetic modification of gene expression that may be activated by the manipulation of the gametes in the laboratory; however, the studies on this topic are scarce (De Rycke *et al*, 2002; Thompson *et al*, 2002; Ayhan *et al*, 2004; Brinton *et al*, 2004).

We review here the cohort studies that have considered the association between ART and cancer in children, and performed a meta-analysis of the available data.

## MATERIALS AND METHODS

Published guidelines for meta-analysis of observational studies (Stroup *et al*, 2000) were followed to perform the literature search and the analysis, and to report the results. Studies included in the present analysis had to meet the following inclusion criteria: they had to be cohort studies involving children born after ART (which represents the exposure of interest), and cancer (all types) had to be the end point. A search on Medline and Embase was performed for articles reported up to February 2005, using combinations of the keywords ‘IVF’, ‘ART’, ‘children’, ‘cohort’ and ‘cancer’, and restricting the search to articles published in English.

A broad search yielded more than 2500 potentially relevant titles. The titles and the abstracts of the papers were screened independently by two experts, and 161 articles which contained information on both short- and long-term health outcomes of children born after ART were selected. Citation indices, bibliographies of the articles and review papers (Brinton *et al*, 2004; Schieve *et al*, 2004; Lightfoot *et al*, 2005) were also checked to complete the search. We selected a total of 14 studies that met the inclusion criteria for the meta-analysis. A description of the studies is reported in Table 1.

Out of the 14 studies, five (Bergh *et al*, 1999; Ericson *et al*, 2002; Pinborg *et al*, 2003, 2004a, 2004b) were partially overlapping, therefore only the two most recent publications, with the larger cohort were included in the meta-analysis (Pinborg *et al*, 2004b for the Danish data set, Ericson *et al*, 2002 for the Swedish data set). The meta-analysis was therefore performed on 11 of the 14 data sets (Table 2), which reported pertinent, nonoverlapping, and comparable data. Three studies have different designs (White *et al*, 1990; Odone-Filho *et al*, 2002; Moll *et al*, 2003), but were included since it was possible to calculate cancer incidence ratios from the available published data; however, a sensitive analysis was conducted by including and excluding these studies.

### Statistical analysis

Two studies (Rufat *et al*, 1994; Pinborg *et al*, 2004b) did not provide cancer incidence rates in a reference population. For the French study (Rufat *et al*, 1994), the expected cases were extracted from the literature (Bernard *et al*, 1993; Gembara *et al*, 1995). Since the average period of follow-up of children born after ART was not reported in the original paper by Rufat, we estimated an average follow-up of 2.2 years, which is the mean between the minimum and the maximum period of follow-up.

For the Danish data sets (Pinborg *et al*, 2003, 2004a, 2004b) the expected number of cases was calculated by applying the cancer incidence rate provided by the Danish Cancer Registry for children 0–6 years and for the period 1995–1999. The average period of follow-up used to calculate the number of expected cases was 4.1 years, as reported in a subsequent publication on the same cohort of children (Lidegaard *et al*, 2005).

For the meta-analysis, the observed and expected cases from each study were added and the overall Standardised Incidence Ratio (SIR) was calculated as the ratio between the number of observed and the number of expected cases. The details for the calculation of the expected number of cases are reported for each study in Table 2. The exact confidence interval for SIR was obtained by using the Poisson's distribution.

The heterogeneity across studies was analysed with the Cochran's test. A SIR adjusted for study was then calculated, using either a fixed or a random-effects model, according to the results of the Cochran's test (Normand, 1999).

The potential for publication bias was examined by drawing a ‘funnel plot’ in which study-specific log effect estimates were plotted against their s.e. (Sterne *et al*, 2000). Egger's test was performed to assess the symmetry of the funnel plot (Begg and Mazumdar, 1994; Egger *et al*, 1997). A significant asymmetry indicates the presence of bias, which was set in this analysis at a *P*-value <0.05. The statistical analysis was performed using STATA package, version 8.

## RESULTS

**Figure 1:**
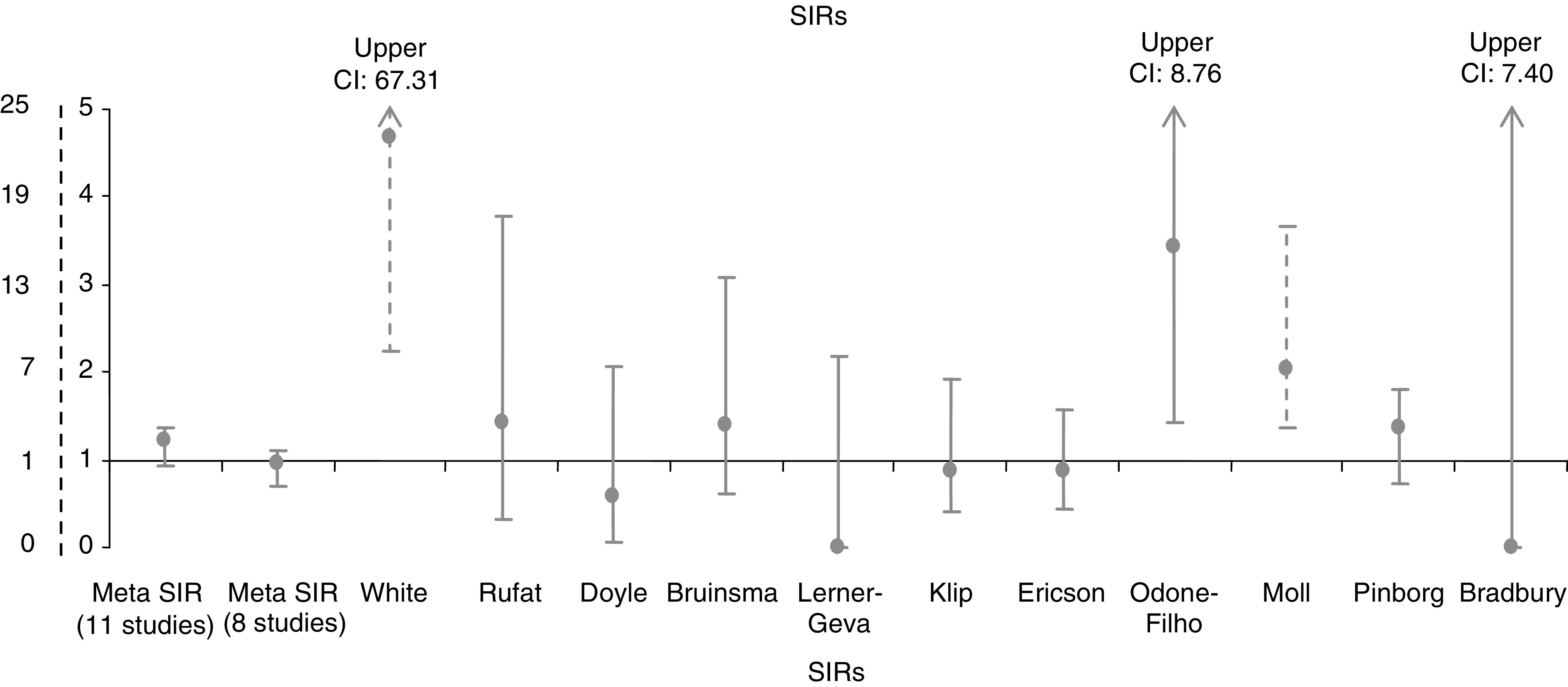
Study specific Standardised Incidence Ratios of cancer in children born after ART. *Notes*: The studies by White and Moll are represented using the dotted *X*-axis scale. The upper CIs for studies by White, Odone-Filho and Bradbury are not graphically represented, and are indicated at the top of each line.

Out of the 11 studies included in this meta-analysis, two were conducted in Australia, six in Europe, one in Israel, one in USA and one in Brasil. Eight were cohort studies, three (White *et al*, 1990; Odone-Filho *et al*, 2002; Moll *et al*, 2003) the last two based on a hypothetical cohort and one based on national statistical data without a systematic follow-up of all the children in the cohort (White *et al*, 1990).The follow-up varied from about 1 to 13 years (Table 1). None of the cohort studies reported a significant association between ART and childhood cancer. The three studies with a different design reported a significant increase of neuroectodermal cancer (White *et al*, 1990), retinoblastoma (Moll *et al*, 2003) or cancer in general (Odone-Filho *et al*, 2002) in children conceived after IVF. The SIR for each study are presented in Figure 1.

The overall assumption of homogeneity between study-specific SIRs was rejected (*P*-value for Cochran's test <0.001), even when restricted to the eight studies with similar design (*P*-value for Cochran's test: 0.003). The lack of homogeneity seemed to be due to two studies (Lerner-Geva *et al*, 2000; Bradbury and Jick, 2004), since the SIR=0 could strongly influence the result even though the studies included a small number of subjects (332 and 176, respectively). When these two studies were excluded from the analysis, the hypothesis of homogeneity could be accepted (*P*-value for Cochran's test: 0.76).

The final cohort included 38 815 subjects, with 38.21 cases of cancer expected *vs* 47 observed, giving a SIR of 1.23 (95% CI 0.93–1.37). The analysis restricted to eight studies (excluding the studies by White, Odone-Filho and Moll, with different designs) indicates 36.22 expected cases of childhood cancer and 35 observed, giving a SIR of 0.97 (95% CI 0.69–1.10).

The study-adjusted SIR was 1.33 (95% CI 0.62–2.85) when all the 11 studies were included, while it was 0.77 (95% CI 0.41–1.42) when the analysis was restricted to eight studies.

The overall analysis did not show publication bias (Egger's test *P*-value: 0.70), while there was evidence of publication bias for the restricted analysis (eight studies, Egger's test *P*-value: 0.02). When the two studies with SIR=0 were excluded, no evidence of publication bias was observed (*P*-value for the Egger's test: 0.40).

## DISCUSSION

Studies on childhood cancer in children born after ART have been conducted only recently, and are analysed in this paper. Overall, no increased risk of childhood cancer was found in the present analysis. A previous review on four of the studies included in this meta-analysis (Lightfoot *et al*, 2005) provided a meta-SIR of 1.03 (95% CI 0.61–1.63).

Meta-analysis is a useful approach when studying rare diseases, such as childhood cancer, because the pooled data set has greater power than each individual study (Egger and Smith, 1997; Blettner *et al*, 1999). However, pooling data can also have certain limitations, (Blettner *et al*, 1999; Stroup *et al*, 2000), such as publication bias. Although our analysis suggests overall a lack of such bias, the number of studies included was small, and therefore our results are not conclusive.

Another issue is that the studies in a meta-analysis may differ considerably in quality, design, methods of data collection, definition of the exposure and type of confounding variables. To promote homogeneity, we included only cohort studies of children followed up for several years. Three studies (White *et al*, 1990; Odone-Filho *et al*, 2002; Moll *et al*, 2003) presented a slightly modified design, so we performed a sensitivity analysis by including and excluding these studies from the metaestimates.

Our meta-analysis could not take into account the length of follow-up as a covariate, since not all the studies included specified it, and when they did, it appeared obvious that the follow-up period was different from study to study.

In this analysis, we concentrated on ART and did not consider studies of the possible negative consequences of hormones administered to the mothers for infertility problems, some of which have included suggestions of (nonsignificant) increases of childhood cancer. Similarly, studies of congenital malformation in relation to ART have not fallen within the scope of our meta-analysis.

In conclusion, this meta-analysis does not suggest an association between ART and childhood cancer, even though the limited number of studies prevent a firm conclusion and a pooled analysis would be useful.

## Figures and Tables

**Table 1:** Cohort studies on *in vitro* fertilization and childhood cancer

**Authors (year)**	**Country**	**Number of exposed children**	**Follow**-**up**	**Cancer results**
White *et al* (1990)	Australia	2285^a^	Absent	Three neuroectodermal tumours
Rufat *et al* (1994)	France	1637	Minimum follow-up: 1 year	One leukaemia
Doyle *et al* (1998)	Britain	2507	Average follow-up: 8.6 years	Two unspecified cancers
Bergh *et al* (1999)	Sweden	5586	Maximum follow-up: 13 years	One ALL, one reticulosis, one upper extremities, one peripheral nerves cancer
Bruinsma *et al* (2000)	Australia	5249	Average follow-up: 3 years, 9 months	One brain, one connective tissue, three leukaemia, one salivary gland cancer
Lerner-Geva *et al* (2000)	Israel	332	710 person years	0 cancers
Klip *et al* (2001)	Netherlands	9479 (429 after hormonal treatment)	Average follow-up: 4.6 years	Three leukemia, four unspecified cancers
Ericson *et al* (2002)	Sweden	9056	Maximum follow-up: 13 years	Three ALL, two histiocytosis, two sarcomas, two CNS, one retinal, one hepatic carcinoma
Odone-Filho *et al* (2002)	Brazil	b	Maximum follow-up: 5 years	One AML, one neuroblastoma, two rhabdo myosarcoma
Moll *et al* (2003)	Netherlands	c	Maximum follow-up: 1 year, 2 months	Five retinoblastoma
Pinborg *et al* (2003)	Denmark	1080 (454 twins)	Maximum follow-up: 4 years	One 1 ALL, one germinal cell tumour
Pinborg *et al* (2004a)	Denmark	3393 (twins)	Minimum follow-up: 1 year	0 cancers
Pinborg *et al* (2004b)	Denmark	8523 (3393 twins)	Minimum follow-up: 1 year	ALL, hepatoblastoma, unspecific tumours of thorax, heart, cerebrum
Bradbury and Jick (2004)	USA	176	Maximum follow-up: 13 years	0 cancers

a Cohort without a systematic follow-up.

b Hypothetical cohort, assuming that approximately 2000 children were conceived after IVF during the period 1996–2000.

c Hypothetical cohort, assuming that 1–1.5% of children were conceived after IVF.

ALL=acute lymphoblastic leukaemia; CNS=central nervous system; AML=acute myelocytic leukaemia.

**Table 2:** Cohort studies included in the meta-analysis

**Author (year)**	**Number of exposed children**	**Calculation of expected cases (EC)**	**Observed/expected cases (*N*)**	**Standardized incidence ratio (95%CI)**
White *et al* (1990)	2285	No calculation in the original study. EC calculated by applying cancer incidence rates from the original study to the IVF cohort	3/0.13	23.08 (8.38–67.31)
Rufat *et al* (1994)	1637	No calculation in the original study. EC calculated by applying published cancer incidence rates (Bernard *et al*, 1993; Gembara *et al*, 1995) to the IVF cohort	1/0.7	1.43 (0.31–3.79)
Doyle *et al* (1998)	2507	Application of cumulative national cancer rates, taking into account year of birth and length of follow-up	2/3.5	0.57 (0.07–2.06)
Bruinsma *et al* (2000)	5249	Application of the Victorian cancer incidence rates, taking into account age and length of follow-up	6/4.33	1.39 (0.62–3.09)
Lerner-Geva *et al* (2000)	332	Application of specific national cancer incidence rates, taking into account age, gender and year of diagnosis	0/1.7	0 (0–2.18)^a^
Klip *et al* (2001)	9050^b^	Application of cancer incidence rates from the Eindhoven and the Netherlands Cancer Registries, taking into account age, gender and calendar period	6/6.78	0.88 (0.41–1.98)
Ericson *et al* (2002)	9056	Application of the Swedish Cancer Registry cancer incidence rates, taking into account year of birth, maternal age, parity and length of involuntary childlessness	11/12.5	0.88 (0.5–1.13)
Odone-Filho *et al* (2002)	Around 2000	Application of annual incidence rate of cancer for children aged 0–4 years	4/1.17	3.42 (1.42–8.76)
Moll *et al* (2003)	Not known	Application of the 1-year age-specific mortality rates from statistics in the Netherlands	5/0.69	7.25 (3.19–17.03)
Pinborg *et al* (2004a, 2004b)	8523	No calculation in the original study. EC calculated by applying the 0–6 years cancer incidence rates from the Danish Cancer Registry to the IVF cohort	9/6.7	1.34 (0.71–1.78)
Bradbury and Jick (2004)	176	No calculation in the original study. EC were calculated by applying retinoblastoma incidence rates from the original study to the IVF cohort	0/0.01	0 (0–7.40)^a^

a The upper confidence limit was calculated using 0.5 as observed number of cases.

b In all, 429 children conceived after hormonal treatment, but not after ART, were excluded.

## References

[bib1] Ayhan A, Salman MC, Celik H, Dursun P, Ozyuncu O, Gultekin M (2004) Association between fertility drugs and gynecologic cancers, breast cancer, and childhood cancers. Acta Obstet Gynecol Scand 83: 1104–1111 15548140 10.1111/j.0001-6349.2004.00669.x

[bib2] Begg CB, Mazumdar M (1994) Operatine chatacteristics of a rank correlation test for publication bias. Biometrics 50: 1088–1101 7786990

[bib3] Bergh T, Ericson A, Hillensjo T, Nygren KG, Wennerholm UB (1999) Deliveries and children born after *in-vitro* fertilisation in Sweden 1982–95: a retrospective cohort study. Lancet 354: 1578–1585 10560671 10.1016/S0140-6736(99)04345-7

[bib4] Bernard JL, Bernard-Couteret E, Coste D, Thyss A, Scheiner C, Perrimond H, Mariani R, Deville A, Michel G, Gentet JC, Raybaud C (1993) Childhood cancer incidence in the south-east of France. A report of the Provence-Alpes-Cote d'Azur and Corsica Regions Pediatric Cancer Registry, 1984–1991. Eur J Cancer 29A: 2284–2291 8110500 10.1016/0959-8049(93)90223-3

[bib5] Blettner M, Sauerbrei W, Schlehofer B, Scheuchenpflug T, Friedenreich C (1999) Traditional reviews, meta-analyses and pooled analyses in epidemiology. Int J Epidem 28: 1–9 10.1093/ije/28.1.1 10195657

[bib6] Bradbury BD, Jick H (2004) *In vitro* fertilization and childhood retinoblastoma. Br J Clin Pharmacol 58: 209–211 15255804 10.1111/j.1365-2125.2004.02109.x PMC1884583

[bib7] Brinton LA, Kruger Kjaer S, Thomsen BL, Sharif HF, Graubard BI, Olsen JH, Bock JE (2004) Childhood tumor risk after treatment with ovulation-stimulating drugs. Fertil Steril 81: 1083–1091 15066468 10.1016/j.fertnstert.2003.08.042

[bib8] Bruinsma F, Venn A, Lancaster P, Speirs A, Healy D (2000) Incidence of cancer in children born after *in-vitro* fertilization. Hum Reprod 15: 604–607 10686204 10.1093/humrep/15.3.604

[bib9] Cruysberg JRM, Moll AC, Imhof SM (2002) Bilateral sporadic retinoblastoma in a child born after *in vitro* fertilization. Arch Ophthalmol 120: 1773 12470166

[bib10] De Rycke M, Liebaers I, Van Steirteghem A (2002) Epigenetic risks related to assisted reproductive technologies: risk analysis and epigenetic inheritance. Hum Reprod 17: 2487–2494 12351517 10.1093/humrep/17.10.2487

[bib11] Doyle P, Bunch KJ, Beral V, Draper GJ (1998) Cancer incidence in children conceived with assisted reproduction technology [letter]. Lancet 352: 452–453 9708757 10.1016/s0140-6736(05)79186-8

[bib12] Egger M, Smith D, Schneider M, Minder C (1997) Bias in meta-analysis detected by a simple, graphical test. BMJ 315: 629–634 9310563 10.1136/bmj.315.7109.629 PMC2127453

[bib13] Egger M, Smith GD (1997) Meta-analysis. Potentials and promise. BMJ 315: 1371–1374 9432250 10.1136/bmj.315.7119.1371 PMC2127866

[bib14] Ericson A, Nygren KG, Otterblad Olausson P, Kallen B (2002) Hospital care utilization of infants born after IVF. Hum Reprod 17: 929–932 11925384 10.1093/humrep/17.4.929

[bib15] Gembara P, Dechelotte P, Chauvin F, Malpuech G, Chazal J, Carla H, Chopard P, Foulon E, Goddon R, Goumy P, Masson A, Labbe A, Deméoca F (1995) Cancers in children in the Auvergne area: retrospective study from 1986 to 1991. Arch Pediatr 2: 622–627 7663649 10.1016/0929-693x(96)81215-2

[bib16] Hansen M, Bower C, Milne E, de Klerk N, Kurinczuk JJ (2005) Assisted reproductive technologies and the risk of birth defects-a systematic review. Hum Reprod 20: 328–338 15567881 10.1093/humrep/deh593

[bib17] Klip H, Burger CW, de Kraker J, van Leeuwen FE (2001) Risk of cancer in the offspring of women who underwent ovarian stimulation for IVF. Hum Reprod 16: 2451–2458 11679537 10.1093/humrep/16.11.2451

[bib18] Lee I, Finger PT, Grifo JA, Rausen AR, Rebarber A, Barad DH (2004) Retinoblastoma in a child conceived by *in vitro* fertilisation. Br J Ophthalmol 88: 1098–1099 15258037 10.1136/bjo.2003.041160 PMC1772288

[bib19] Lerner-Geva L, Toren A, Chetrit A, Modan B, Mandel M, Rechavi G, Dor J (2000) The risk for cancer among children of women who underwent *in vitro* fertilization. Cancer 88: 2845–2847 10870070 10.1002/1097-0142(20000615)88:12<2845::aid-cncr26>3.0.co;2-e

[bib20] Lidegaard O, Pinborg A, Andersen AN (2005) Imprinting diseases and IVF: Danish National IVF cohort study. Hum Reprod 20: 950–954 15665017 10.1093/humrep/deh714

[bib21] Lightfoot T, Bunch K, Ansell P, Murphy M (2005) Ovulation induction, assisted conception and childhood cancer. Eur J Cancer 41: 715–724 15763647 10.1016/j.ejca.2004.07.032

[bib22] Moll AC, Imhof SM, Cruysberg JRM, Schouten-van Meeteren AYN, Boers M, van Leeuwen FE (2003) Incidence of retinoblastoma in children born after *in-vitro* fertilisation. Lancet 361: 309–310 12559867 10.1016/S0140-6736(03)12332-X

[bib23] Normand SL (1999) Meta-analysis: formulating, evaluating, combinig, and reporting. Stat Med 18: 321–359 10070677 10.1002/(sici)1097-0258(19990215)18:3<321::aid-sim28>3.0.co;2-p

[bib24] Odone-Filho V, Cristofani LM, Bonassa EA, Braga PE, Eluf-Neto J (2002) *In vitro* fertilization and childhood cancer. J Pediatr Hematol Oncol 24: 421–422 10.1097/00043426-200206000-00023 12142799

[bib25] Pinborg A, Loft A, Rasmussen S, Schmidt L, Langhoff-Roos J, Greisen G, Andersen AN (2004a) Neonatal outcome in a Danish national cohort of 3438 IVF/ICSI and 10,362 non-IVF/ICSI twins born between 1995 and 2000. Hum Reprod 19: 435–441 14747193 10.1093/humrep/deh063

[bib26] Pinborg A, Loft A, Nyboe Andersen A (2004b) Neonatal outcome in a Danish national cohort of 8602 children born after *in vitro* fertilization or intracytoplasmic sperm injection: the role of twin pregnancy. Acta Obstet Gynecol Scand 83: 1071–1078 15488125 10.1111/j.0001-6349.2004.00476.x

[bib27] Pinborg A, Loft A, Schmidt L, Andersen AN (2003) Morbidity in a Danish national cohort of 472 IVF/ICSI twins, 1132 non-IVF/ICSI twins and 634 IVF/ICSI singletons: health-related and social implications for the children and their families. Hum Reprod 18: 1234–1243 12773452 10.1093/humrep/deg257

[bib28] Rizk T, Nabbout R, Koussa S, Akatcherian C (2000) Congenital brain tumor in a neonate conceived by *in vitro* fertilization. Child's Nerv Syst 16: 501–502 11007501 10.1007/PL00007295

[bib29] Rufat P, Olivennes F, De Mouzon J, Dehan M, Frydman R (1994) Task force report on the outcome of pregnancies and children conceived by *in-vitro* fertilization (France: 1987–1989). Fertil Steril 61: 324–330 8299791

[bib30] Schieve LA, Rasmussen SA, Buck GM, Schendel DE, Reynolds MA, Wright VC (2004) Are children born after assisted reproductive technology at increased risk for adverse health outcomes? Obstet Gynecol 103: 1154–1163 15172847 10.1097/01.AOG.0000124571.04890.67

[bib31] Sterne JA, Gavaghan D, Egger M (2000) Publication and related bias in meta-analysis: power of statistical tests and prevalence in the literature. J Clin Epidemiol 53: 1119–1129 11106885 10.1016/s0895-4356(00)00242-0

[bib32] Stroup DF, Berlin JA, Morton SC, Olkin I, Williamson GD, Rennie D, Moher D, Becker BJ, Sipe TA, Thacker SB (2000) Meta-analysis of observational studies in epidemiology: a proposal for reporting. Meta-analysis Of Observational Studies in Epidemiology (MOOSE) group. JAMA 283: 2008–2012 10789670 10.1001/jama.283.15.2008

[bib33] Thompson JG, Kind KL, Roberts CT, Robertson SA, Robinson JS (2002) Epigenetic risks related to assisted reproductive technologies: risk analysis and epigenetic inheritance. Hum Reprod 17: 2487–2494 12351517 10.1093/humrep/17.10.2487

[bib34] Toren A, Sharon N, Mandel M, Neumann Y, Kenet G, Kaplinsky C, Dor J, Rechavi G (1995) Two embryonal cancers after *in vitro* fertilization. Cancer 76: 2372–2374 8635045 10.1002/1097-0142(19951201)76:11<2372::aid-cncr2820761128>3.0.co;2-o

[bib35] White L, Giri N, Vowels MR (1990) Neuroectodermal tumours in children born after assisted conception [letter]. Lancet 336: 1557 1979385 10.1016/0140-6736(90)93350-x

